# The analysis of cathepsin L that mediates cellular SARS‐CoV‐2 infection leading to COVID‐19 in head and neck squamous cell carcinoma

**DOI:** 10.3389/fimmu.2023.1156038

**Published:** 2023-05-23

**Authors:** Feng Gao, Xia Wang, Nianhong Qin, Mingxia Zhang, Mingfeng Liao, Meiqi Zeng, Desheng Lu, Ou Sha

**Affiliations:** ^1^School of Dentistry, Institute of Stomatological Research, Medical School, Shenzhen University, Shenzhen, China; ^2^Medical School, Shenzhen University, Shenzhen, China; ^3^Institute for Hepatology, National Clinical Research Center for Infectious Disease, Shenzhen Third People’s Hospital, Shenzhen, China

**Keywords:** cathepsin L, head and neck squamous cell carcinoma (HNSCC), immunotherapy, CTSL, SARS-CoV-2

## Abstract

The vulnerability of the oral cavity to SARS-CoV-2 infection is well-known, and cancer patients are at a higher risk of COVID-19, emphasizing the need to prioritize this patient population. Head and neck squamous cell carcinoma (HNSCC) is one of the most common malignant cancers associated with early metastasis and poor prognosis. It has been established that cancerous tissues express Cathepsin L (CTSL), a proteinase that regulates cancer progression and SARS-CoV-2 entry. Therefore, it is essential to evaluate the correlation between disease outcomes and CTSL expression in cancer tissues and predict the susceptibility of cancer patients to SARS-CoV-2. In this study, we used transcriptomic and genomic data to profile CTSL expression in HNSCC and developed a CTSL signature that could reflect the response of HNSCC patients to chemotherapy and immunotherapy. Additionally, we investigated the relationship between CTSL expression and immune cell infiltration and established CTSL as a potential carcinogenic factor for HNSCC patients. These findings could aid in understanding the mechanisms underlying the increased susceptibility of HNSCC patients to SARS-CoV-2 and contribute to the development of therapy for both HNSCC and COVID-19.

## 1 Introduction

The emergence of the novel coronavirus (2019-Nov) in December 2019 has resulted in a global spread, leading to large-scale pandemics and posing a significant threat to public health and the economy (1). COVID-19 has become a widespread and deadly disease, with a rapid increase in infections and fatalities reported worldwide (1). Although most people recover, there is concern that some may experience devastating consequences, as the long-term effects of the virus are still uncertain. To effectively control SARS-CoV-2, it is necessary to prevent infection and conduct further research to determine the factors that influence susceptibility to COVID-19 and the mechanisms behind these factors. SARS-spike CoV-2’s glycoprotein has been shown to bind the angiotensin-converting enzyme 2(ACE2), speeding up the virus’s entrance into host cells (2, 3). After binding to the target cells, TMPRSS2 and host cell proteases such as cathepsin L (CTSL) split the S protein into two subunits: S1 and S2, facilitating viral entry into the host cells by promoting membrane fusion and endocytosis of coronaviruses. S protein cleavage by host proteases is required for viral activation and subsequent infection (3, 4). As a result, differences in susceptibility to SARS-CoV-2 infection may be explained by CTSL expression and distribution to a certain extent.

Current evidence suggests that SARS-CoV-2 infection causes considerable morbidity and mortality. Some studies revealed that the oral cavity is one of the areas most vulnerable to SARS-CoV-2 (1), with oral manifestations possibly presenting before other COVID-19 manifestations. According to recent studies, cancer patients reportedly experience a higher incidence of COVID-19, more severe symptoms, and a poor prognosis (5). Accordingly, more emphasis should be placed on preventing COVID-19 infection in cancer patients.

Tumor pathogenesis is a complex process involving multiple pathophysiological processes and is impacted by numerous factors, including the body’s immune status. The role of cellular and humoral immunity in viral infection protection is well established. The pathogenesis of COVID-19 is widely believed to be linked to immune response dysregulation, especially in T cells (6). Merad et al. reported that COVID-19 pathogenesis was mediated by aberrant and numerous immune cells, such as monocytes and macrophages (7). Besides, anticancer therapy, such as chemotherapeutics or radiation, can cause systemic immunosuppression (5). It is widely thought that cancer patients are more susceptible to SARS-CoV-2 infection due to immune dysregulation (8, 9). The expression of SARS-CoV2 receptors (ACE2, TMPRSS2, and CTSL) has been reported to be upregulated in many types of malignancies, making viral entry into cells easier and cancer patients more susceptible to SARS-CoV-2 (10).

Herein, we aimed to better understand the relationship between SARS-CoV-2 and head and neck squamous cell carcinoma (HNSCC). We utilized transcriptomic and genomic data to conduct a systematic analysis of the role of CTSL in HNSCC, focusing on immunological characteristics, functional annotation, and prediction of chemotherapy response. Furthermore, we developed a novel CTSL-related signature that could effectively predict HNSCC patients’ outcomes. The current study substantiated that CTSL is a potentially carcinogenic factor for patients with HNSCC, which helps researchers better understand the increased susceptibility of HNSCC to SARS-CoV-2 infection and lays the groundwork for SARS-CoV-2 therapy.

## 2 Materials and methods

### 2.1 HNSC dataset and preprocessing

We acquired genomic data and annotated clinicopathological features of HNSC from The Cancer Genome Atlas (TCGA, 564 HNSC patients, including 520 tumors and 44 paracancers, and 519 tumor patients with complete survival information) and Gene Expression Omnibus (GEO) (datasets GSE41613, N=97 and GSE65858, N=270). The study included only cases with adequate OS information, while cases with insufficient information were excluded.

Raw data from the GEO database were generated using Affymetrix and Illumina platforms. The data were background corrected and normalized using a robust multichip averaging algorithm (RMA). RNA sequencing data were obtained from the TCGA database and converted from fragments to transcripts with signal intensities, similar to the data obtained from RMA.

### 2.2 Establishment of the CTSL-based Signature

High and low-expression groups were classified according to CTSL expression in TCGA-HNSC and underwent genome-wide difference analysis using the package R “limma” (abs(logFC) > 1 & P.Value< 0.01). Subsequently, Cox regression analysis was performed to further identify prognosis-related CTSL-associated genes (R language “survival” package, P<0.05). We then applied a survival machine learning algorithm *via* the R package “randomSurvivalForest” to screen significant valuable CTSL-associated genes with prognostic potential. Based on the Lasso regression analysis, the CTSL-based signature was constructed using the list of prognosis-related CTSL genes (11).

### 2.3 Validation of the efficacy of CTSL-based signature

We applied the CTSL-based signature to the data of 516 patients from the TCGA-HNSC dataset and then divided the patients into high and low CTSL. The clustering of signatures was based on the P value of the optimal cutoff, and the relationship between the CTSL-based signature and OS was analyzed using Kaplan-Meier curves. The performance of the CTSL-based signature for predicting prognosis at 1, 3, and 5 years were evaluated using TimeROC.

### 2.4 Genomic alteration

The TCGA datasets were used to collect somatic mutations. Somatic mutation analysis was achieved with the R package “maftools”.

### 2.5 TME immunological profile assessment

The study utilized the Estimation of STromal and Immune cells in MAlignant Tumor tissues using Expression data (ESTIMATE) technique to evaluate the abundance of immune cells and the level of stromal cell infiltration. This technique generates immune scores, stromal scores, and estimated scores to represent these factors. To analyze immune infiltrating cells in HNSC, we used the Tumor Immune Estimation Resource 2.0 (TIMER2.0; http://timer.cistrome.org) web server. The R genomic variance analysis package (GSVA) was used in conjunction with single sample genomic enrichment analysis to construct an enrichment score that represented the degree of infiltration of 28 immune cells based on the associated characteristics (ssGSEA).

### 2.6 Functional annotation

All gene sets were obtained from the MSigDB database, the Kyoto Encyclopedia of Genes and Genomes (KEGG), and Gene Ontology (GO). Gene set enrichment analysis (GSEA) and genomic variation analysis (GSVA) were implemented using the R packages clusterProfiler and GSVA.

### 2.7 Drug response prediction

To predict drug susceptibility in the cases included, the researchers used pharmacogenomic data from the Genomics of Drug Sensitivity in Cancer (GDSC) database (https://www.cancerrxgene.org/). They calculated drug response as drug susceptibility using the oncoPredict R package. The responses to anti-PD1 and anti-CTLA4 therapies in HNSC were evaluated by the submap algorithm.

### 2.8 CCK8 assay

To evaluate cell proliferation, the transfected cells were seeded into 96-well plates. After being cultured at 37°C with 5% CO2 for 24, 48, and 72 hours, 20 L of CCK8 solution (Sigma, USA) was added to each well, and the plates were incubated for an additional hour. Using a microplate reader, the optical density (OD) value obtained at 560nm wavelength was determined (Molecular Devices, Sunnyvale, CA).

### 2.9 Plate clone formation assay

To validate the ability of the transfected cells to form colonies, 1000 cells per plate were seeded in 60mm culture dishes. After being cultured for 10 days, the cells were fixed with 10% neutral buffer formalin fixative and stained with crystal violet (Beyotime, China). We photographed and counted the colonies.

### 2.10 Transwell assay

Transfected cells were seeded onto transwell membranes coated with Matrigel (BD Bioscience, Franklin Lakes, NJ) with a solution containing 10% bovine serum albumin (BSA, VWR, Radnor, PA) in the upper chamber (8μm pore size; Corning, Corning, NJ). The lower chamber contained medium with 10% FBS. After incubation at 37°C for 48 hours under 5% CO2, the remaining cells on the top membrane were removed using a cotton swab and fixed in 10% neutral buffer formalin. Crystal violet solution was added before capturing the membranes on camera with an inverted microscope and counting the cells.

### 2.11 Scratch migration assay

6 well plates were inoculated with transfected cells to determine cell migration. After 24 h of cell culture, a straight line wound was created in each well using a pipette tip. The cells were cultured in 2% FBS medium under 5% CO_2_ at 37°C. Wound closure was assessed under 4× magnification with microscopy (OLYMPUS, Tokyo, Japan) at 24 and 48 h.

### 2.12 Construction of lentiviral vectors and infection of lentivirus infection

Using green fluorescent protein (GFP) containing lentiviral, high transfection efficiency and stable CTSL expression were achieved in SCC15 cells. A recombined EX-A4513-Lv201 vector with the CTSL gene and EX-NEG-Lv201 with a negative control sequence was constructed by GeneCopoeia Company (Guangzhou, China). The aforementioned lentiviral vectors were then used to infect SCC15 cells. A total of 1× 10^6^ SCC15 cells were seeded in a six-well cell plate, cultured for an additional 12 hours until 70% confluence was reached, and then lentiviral vectors at a multiplicity of infection (MOI) of 20 units per cell were added to the infection medium. Three groups were established as follows: SCC15 CTSL (CTSL overexpression group), SCC15 NEG (negative group), and SCC15 (non-treatment control group). After incubation for 24 hours, fresh, virus-free media was applied to the plates. After three days, the lentivirus density containing GFP was detected to evaluate the infection efficiency.

### 2.13 Statistical analysis

The Wilcoxon test was utilized for group comparisons when the data were not normally distributed, while the T-test was used when the variables were normally distributed. The difference in OS between the two groups was calculated using Kaplan-Meier survival plots with the R package “survminer”. Cox regression for survival analysis was performed using the R package “survival”. Time-dependent ROC curves were plotted using the R package “timeROC”. Heat maps were generated using the R package “pheatmap”. Data visualization was done using the R package ggplot2 (v). Statistical significance was set at a p-value of 0.05.

## 3 Results

### 3.1 Features associated with CTSL expression in HNSCC

**Figure 1:**
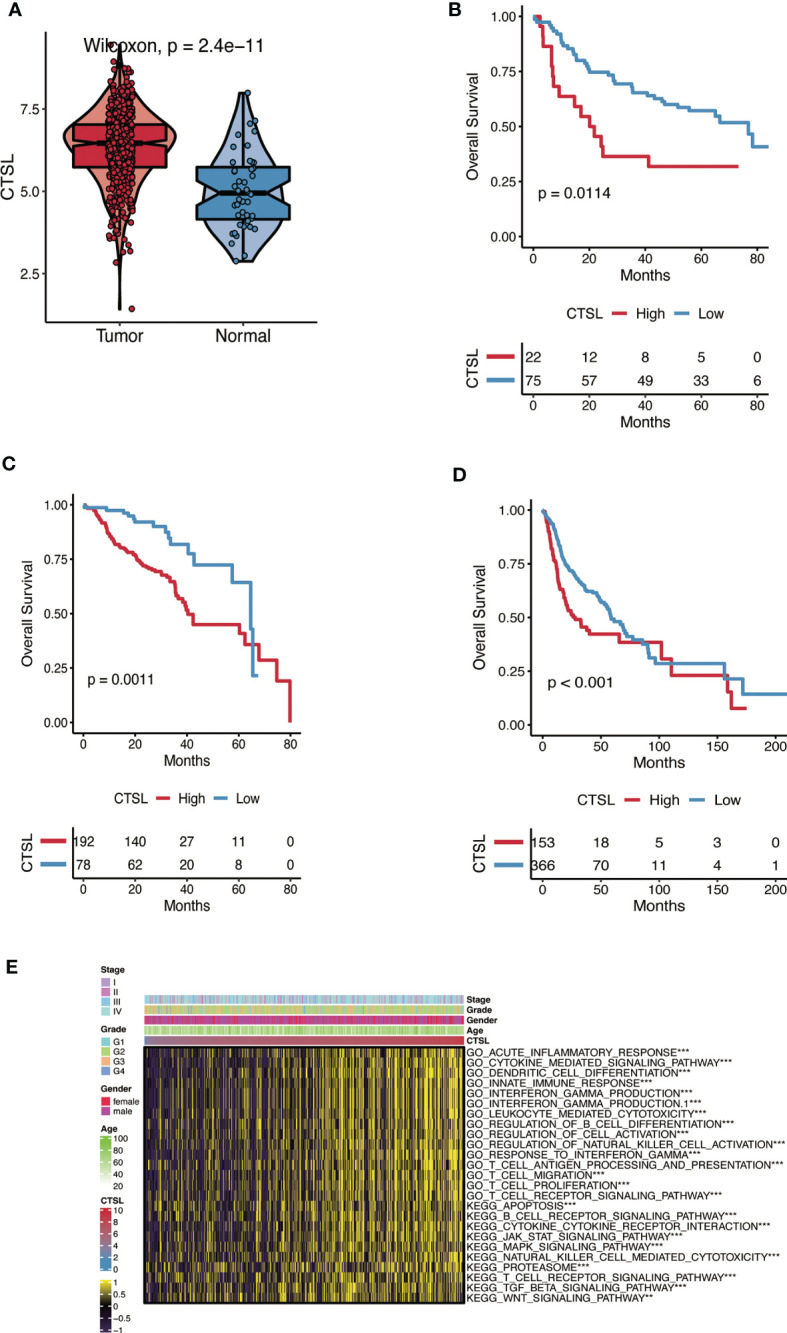
The CTSL expression patterns in HNSC. **(A)** The expression levels of CTSL in HNSC tumor tissues are higher than in normal tissues analyzed in the TCGA dataset (P< 0.01). **(B, C, D)** The association of CTSL expression with overall survival (OS) in HNSC patients (P< 0.01) in 3 databases (GSE41613, GSE65858 and TCGA). **(E)** Functional annotation associated with CTSL in TCGA-HNSC by GSVA analysis. ***, p<0.005, **, p<0.01.

Analysis of the TCGA database revealed that the levels of CTSL mRNA in HNSC were significantly increased compared with adjacent tissues (**Figure 1A**).

The Kaplan-Meier method was used to assess the impact of CTSL expression on the survival of HNSC patients in three datasets (GSE41613, GSE65858, and TCGA). Patients with HNSC who exhibited high levels of CTSL expression were associated with low overall survival (OS) (**Figures 1B-D**). High levels of CTSL transcription have been identified as a significant risk factor for mortality in various cancer types, including HNSC, indicating a poor prognosis. Therefore, CTSL has huge potential as a robust prognostic marker for OS, even after accounting for other relevant variables. Next, GO and KEGG analyses were used to predict CTSL function and associated signaling pathways. GSEA was used to identify the signaling pathways linked with CTSL activated in HNSC.

CTSL was significantly enriched in signaling pathways, including immune response-associated activities such as acute inflammatory response, cytokine-mediated signaling pathway, dendritic cell differentiation, innate immune response, interferon-gamma production, leukocyte mediated cytotoxicity, regulation of B cell differentiation (**Figure 1E**). The GSEA results also showed that several immune functioning gene sets, including apoptosis, B cell receptor signaling, cytokine receptor interaction, and JAK-STAT signaling pathway, were enriched in HNSC. These findings suggest that CTSL plays an essential role in the tumor microenvironment.

### 3.2 CTSL is associated with immune infiltration in HNSCC patients in the TCGA cohort

**Figure 2:**
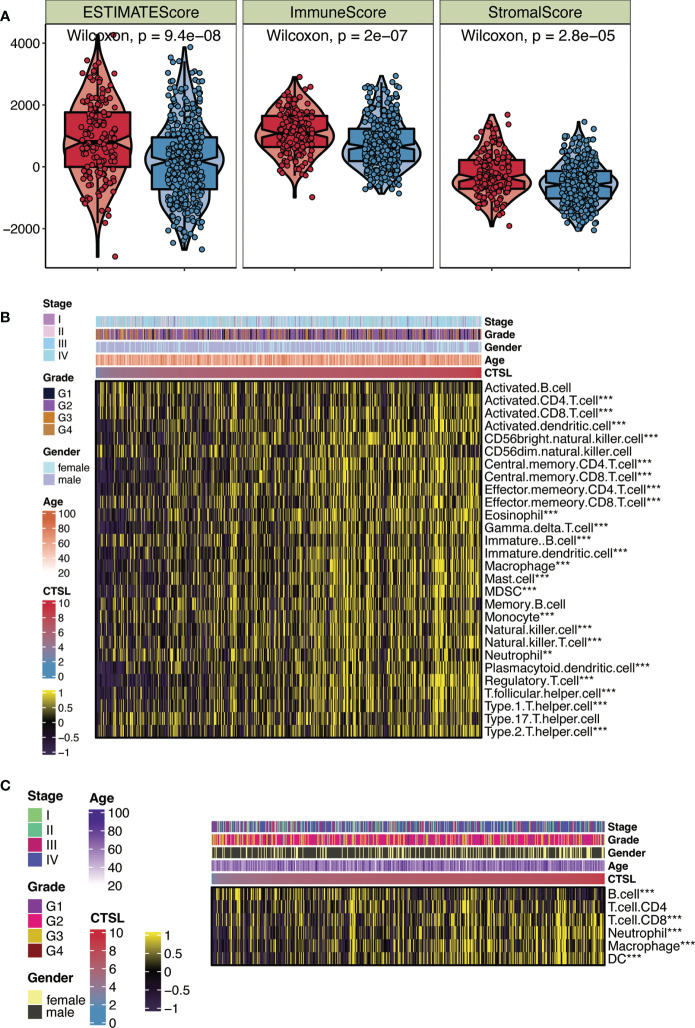
CTSL expression in relation to immunity in the TCGA cohort. **(A)** Changes in ESTIMATE among HNSCC patients with high and low CTSL expression. **(B)** Heatmap showing the abundance of infiltrating immune cell populations for different CTSL expression according tossGSEA algorithms. **(C)** Heatmap showing the abundance of infiltrating immune cell populations for different CTSL expression according to TIMER algorithms. **, p<0.005; ***, p<0.001.

Using the ESTIMATE algorithm, we examined how the immune status of HNSCC patients in the TCGA cohort is associated with the expression levels of CTSL. We discovered that patients with high CTSL expression levels were significantly more likely to have high immune scores, ESTIMATE scores, and stromal cells than patients with low CTSL expression levels (**Figure 2A**), suggesting that CTSL expression levels in HNSCC patients are correlated with immune status.

We independently applied the ssGSEA and TIMER algorithms (**Figures 2B, C**) to uncover the number of immune infiltrating cell groups to validate this characteristic. The generated heat map showed significant infiltration of NK cells, neutrophils, CD8+ T cells, and cytotoxic lymphocytes in HNSCC patients with elevated CTSL expression (**Figure 2B**). We discovered that groups with high CTSL expression had a larger abundance of infiltrating B cells and CD4+ T cells in HNSC (**Figure 2C**). Our findings suggest a significant correlation between the immune response to tumors and the expression levels of CTSL in HNSCC patients. We utilized a heatmap of clinical stages, grade, gender, and age to illustrate the relationship between CTSL expression and various clinical traits of HNSCC samples (**Figures 2B, C**). The findings revealed a significant association between CTSL expression and the clinical traits of HNSCC patients.

### 3.3 Potential immunotherapy and chemotherapy responses associated with CTSL expression in patients with HNSCC

**Figure 3:**
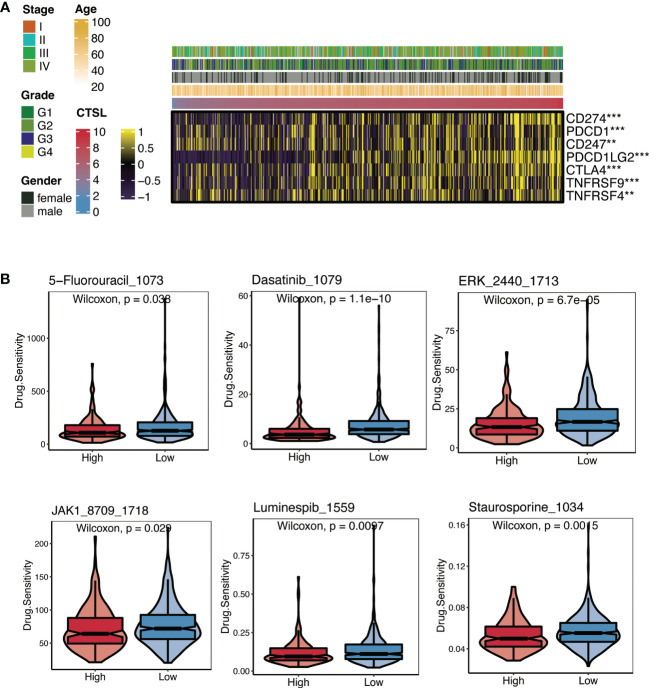
Immunotherapy and chemotherapy of CTSL expression involved in TCGA-HNSCC. **(A)** Correlation of CTSL expression and immune checkpoint levels in HNSCC. **(B)** Boxplots of estimated drug sensitivities for several GDSC chemotherapeutics in the high and low CTSL expression groups. **, p<0.005; ***, p<0.001.

Recent immunotherapy advancements, particularly PD-1 inhibitors, have improved treatment outcomes for HNSCC in the recurrent and metastatic stages. This improvement is caused by the interaction of immune cell processes (12). We first examined the relationship between CTSL expression and immune checkpoint levels in patients with HNSCC to investigate the therapy responsiveness depending on CTSL expression. Association analysis revealed that CD274 and CTLA4 levels were generally higher in HNSCC patients with elevated CTSL expression (**Figure 3A**). In addition, there was a significant association between CTSL expression and targeted therapies (including 5-fluorouracil, dasatinib, ERK_2440, JAK1_8709, luminespib, and staurosporine), indicating that patients with low CTSL expression responded better to targeted therapies (**Figure 3B**).

The results of the submap showed that the high- and low-CTSL groups had different responses to immunotherapy in that the high-CTSL group had a significant response to anti-PD-1 immunotherapy in HNSC based on the TCGA (**Supplementary Figure**).

### 3.4 The CTSL gene serves as an oncogene in HNSC cells

**Figure 4:**
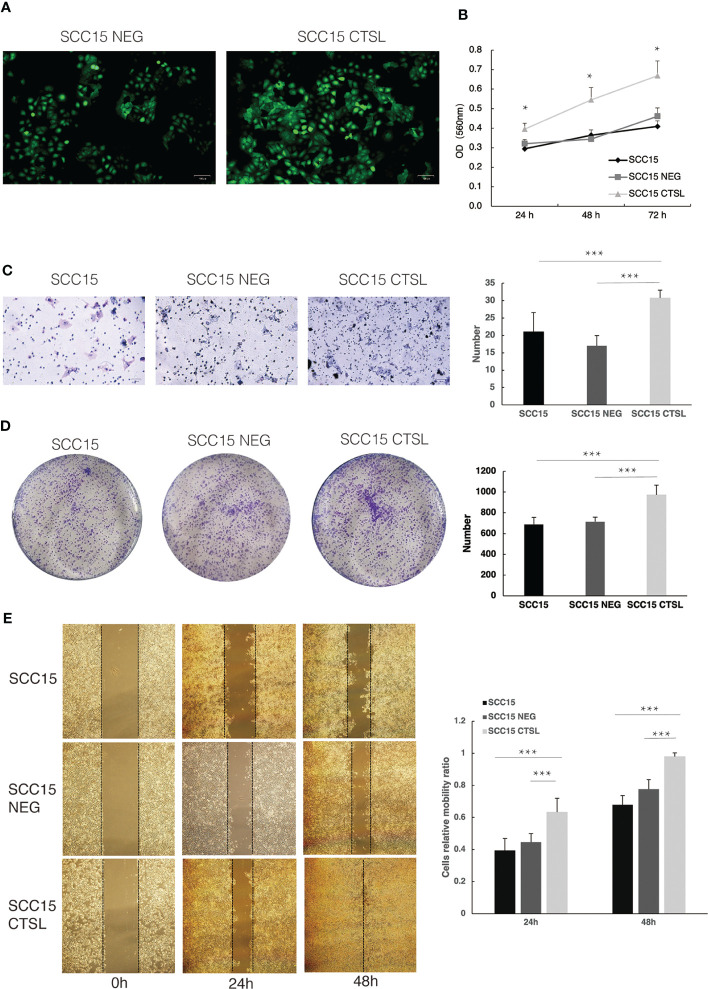
CTSL promotes tumor cell proliferation and metastasis in HNSC. **(A)**Stable transfection of lentivirus with negative control (NEG) or CTSL in SCC15 cells is indicated by green fluorescence (200×). **(B)**CCK8 assay in SCC15 cells, SCC15 NEG cells and SCC15 CTSL cells. **(C)** Transwell assay and its quantitative analysis in SCC15 cells, SCC15 NEG cells and SCC15 CTSL cells (200×). **(D)** Plate cloning assay and its quantitative analysis in SCC15 cells, SCC15 NEG cells and SCC15 CTSL cells. **(E)** scratch migration assay and its quantitative analysis in SCC15 cells, SCC15 NEG cells and SCC15 CTSL cells(40×). *, p<0.05; ***, p<0.01.

We sought to investigate the oncogenic potential of CTSL in HNSC by introducing it into SCC15 cells *via* lentiviral vectors containing GFP. This approach was chosen to achieve high transfection efficiency and ensure stable expression of CTSL. After three days of infection, we used fluorescence microscopy to confirm GFP expression (**Figure 4A**). Our functional assays revealed that CTSL overexpression promoted cell proliferation (CCK8, **Figure 4B**), invasion (Transwell, **Figure 4C**), colony formation (Plate cloning, **Figure 4D**), and migration (scratch migration, **Figure 4E**) in SCC15 cells. These findings collectively support the involvement of CTSL in the progression of HNSC.

### 3.5 The process of constructing CTSL.signature in HNSCC

**Figure 5:**
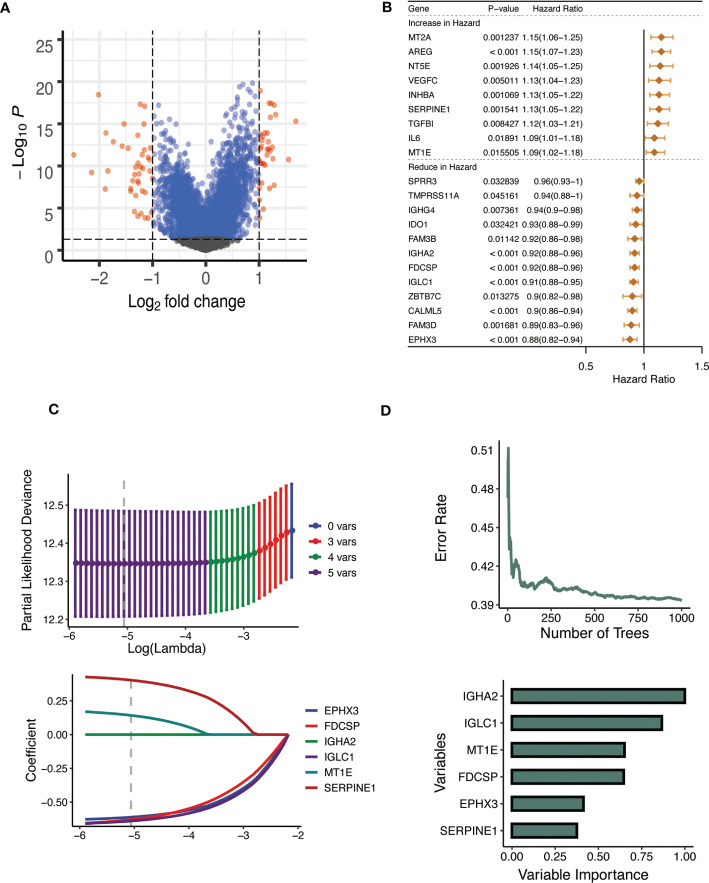
Establishment of the CTSL signature. **(A)** Volcano plot showing the results of the analysis of differences between high and low CTSL groups. **(B)** Univariate Cox analysis forest plot of 21 prognosis-related to CTSL. **(C)** Machine learning method for survival random forest to further screen CTSL signature). **(D)** Lasso regression method to calculate CTSL signature.

After examining the differences between the high and low CTSL groups (**Figure 5A**), we performed a univariate Cox regression analysis to further screen out more valuable CTSL-associated prognostic genes (P value< 0.05). 21 additional CTSL-associated genes with potential prognostic value for HNSCC patients were identified. A forest plot was generated to visualize the HR of each gene on prognosis (**Figure 5B**). In addition, we further selected 9 CTSL-associated genes by applying the machine learning algorithm of (**Figure 5C**). The random survival forest model was used to screen out six genes prognostically associated with CTSL (**Figure 5C**). Finally, Lasso regression analysis was used to calculate new scores based on the estimated regression coefficients of these five prognostic and CTSL-associated genes (**Figure 5D**).

The prognostic CTSL-based signature was as follows: -0.6402∗IGLC1+0.142∗MT1E-0.6298∗FDCSP-0.6116∗EPHX3+0.4029∗SERPINE1 .

### 3.6 Validating the predictive value of CTSL-based signature for HNSCC survival

**Figure 6:**
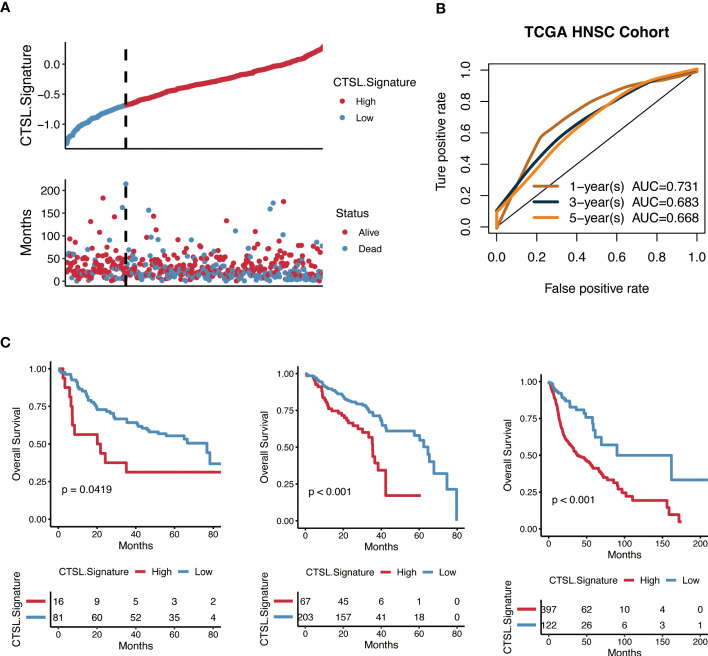
Predictive potential of the CTSL signature for prognosis in HNSCC patients. **(A)** Kaplan–Meier curves of high and low CTSL signature in TCGA-HNSCC. **(B)** Time-dependent ROC of CTSL signature in TCGA. **(C)** Kaplan–Meier curves of overall survival in HNSCC patients based on external validation datasets (GSE41613, GSE65858, and TCGA).

Kaplan-Meier analysis was carried out to determine whether the CTSL-based signature accurately predicted the clinical traits of HNSCC patients. Higher CTSL-based signature scores for HNSCC patients were associated with worse survival curves (**Figure 6A**). The AUC values for the time-dependent ROC curves of the 1-year, 3-year, and 5-year OS were 0.685, 0.712, and 0.746, respectively (**Figure 6B**). This finding suggests that our CTSL signature has prognostic significance. In addition, we further validated the prognostic and survival disadvantage of HNSCC patients with higher CTSL-based signature scores using three independent cohorts (GSE41613, GSE65858, and TCGA) (**Figure 6C**).

### 3.7 CTSL signature expression was associated with genomic alterations

**Figure 7:**
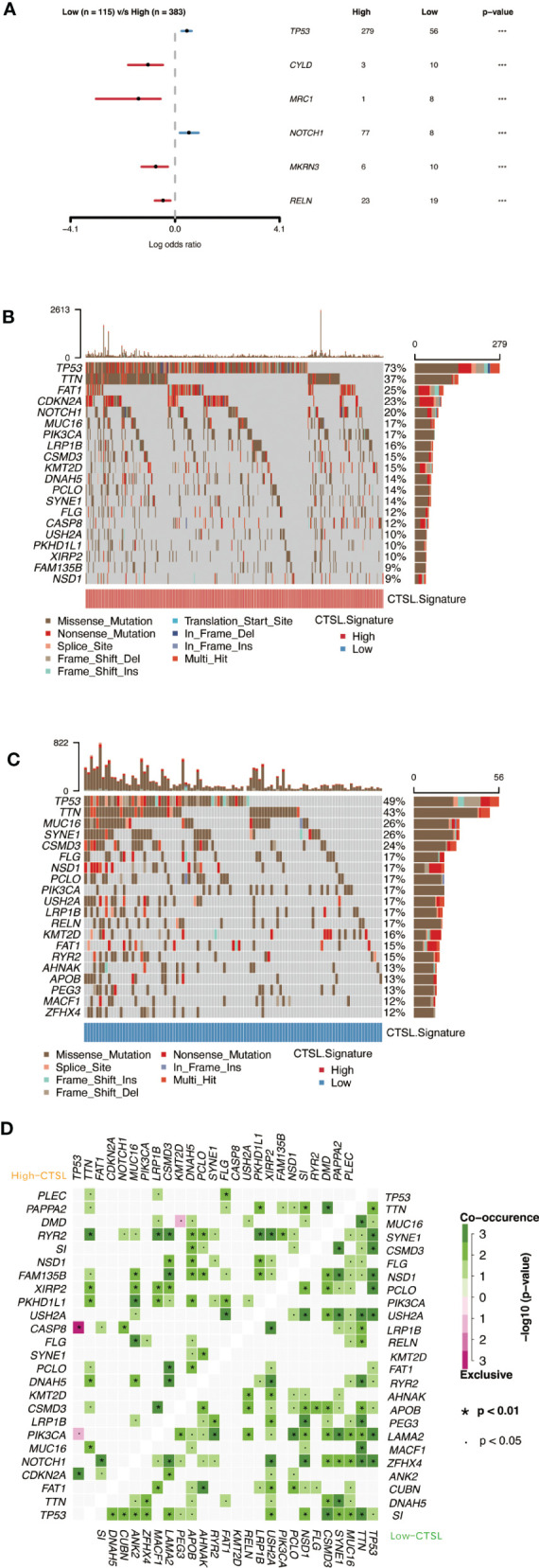
Genomic alterations associated with CTSL signature in HNSCC samples. **(A)** Forest plot showing the results of somatic mutation difference analysis between high and low groups of CTSL signature. **(B, C)** oncoplot of somatic mutations in HNSCCs between high and low CTSL signature groups. **(D)** The heatmap presents the somatic interaction of HNSCCs between CTSL signature high and low groups.

Cellular tumor antigen p53 (TP53) mutation was found to be significantly enriched in both the high-CTSL group and low-CTSL group (73% and 49%, respectively) according to the mutational distribution study (**Figure 7B**), followed by titin (TTN) (37%), (FAT1) (25%), and (CDKN2A) (23%) in the high-CTSL group, and TTN (49%), MUC16 (43%), SYNE1 (26%) in the low-CTSL group (**Figure 7C**).

Furthermore, we used Fisher’s exact test to determine the ratio of mutation frequencies between the high and low CTSL groups and ranked the results based on increasing p-values. The high CTSL group showed higher TP53 and NOTCH1 mutation load while exhibiting lower mutation loadings of CYLD, MRC1, MKRN3, and RELN compared to the low CTSL group (**Figure 7A**). In addition, the concurrent or mutually exclusive mutations in the 25 most frequently mutated genes are shown in **Figure 7D**. The high CTSL group had significantly more concurrent gene alterations than the low CTSL group. notch1 mutations frequently occurred concurrently with FAT1 mutations in the high CTSL group. Other intimate mutant loci included RYR2 and TTN, RYR2 and LRP1B, CSMD3, and RYR2. Common co-mutations in the low CTSL group included NSD1 and USH2A, LAMA2, and TP53. Meanwhile, dense mutually exclusive gene alteration pairs were identified, such as TP53-CASP8 in the high CTSL group (**Figure 7D**).

### 3.8 CTSL-based signature is correlated with immune status in TCGA cohorts with HNSCC

**Figure 8:**
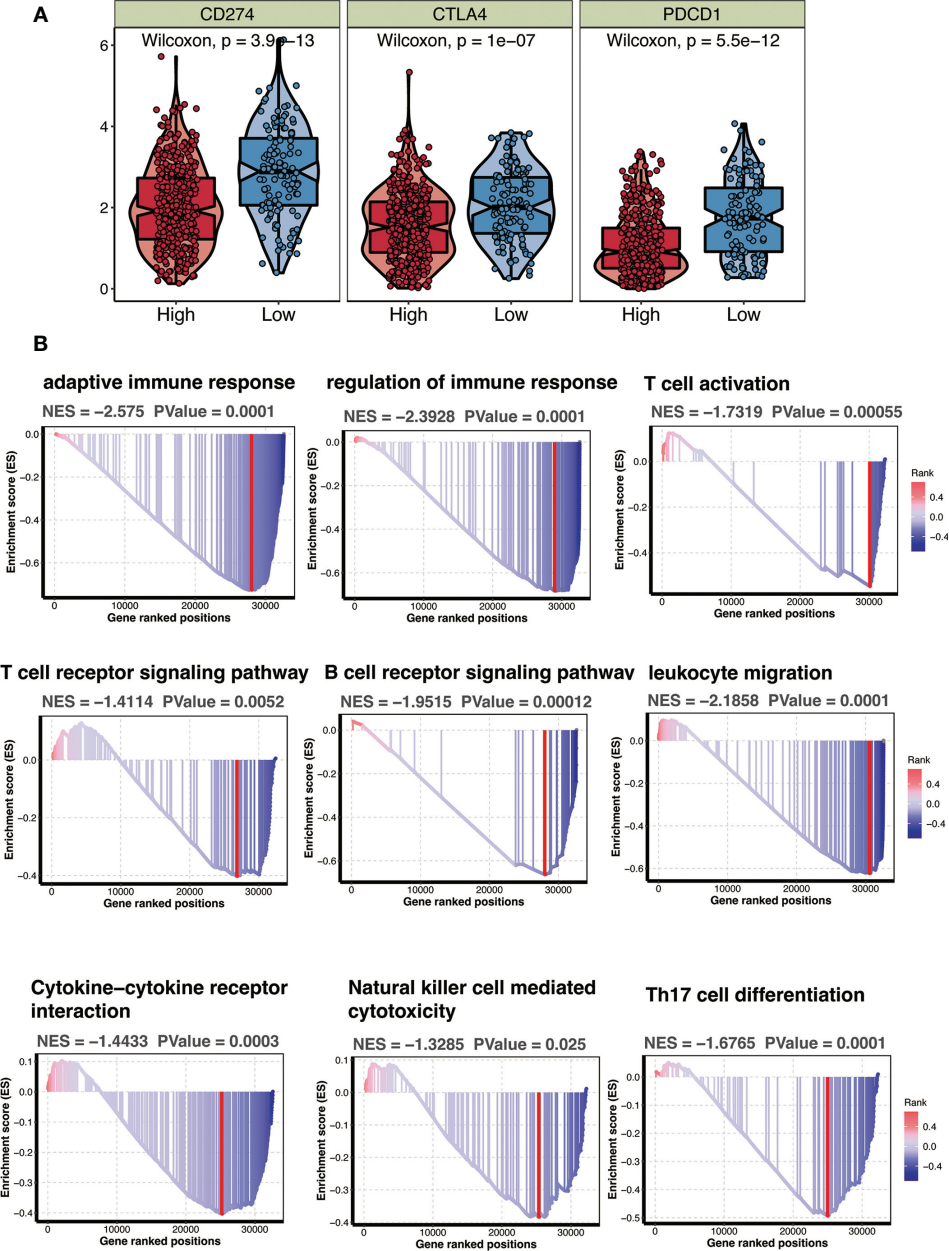
CTSL signature in relation to immunity in the TCGA cohort. **(A)** Changes in ESTIMATEclassical immune checkpoints among HNSCC patients with high and low CTSL signature. **(B)** GSEA showing immune related pathways potentially related by CTSL signature.

To examine the relationship between CTSL-based signature and immune status in TCGA patients with HNSCC, immune checkpoint expression levels were observed to be considerably higher in HNSCC patients with low-CTSL-based signature scores than in those with high-CTSL-based signature scores (**Figure 8A**), demonstrating that in HNSCC patients, the levels of CTSL signature scores were inversely linked with immunological state. Furthermore, GSEA analysis found that HNSCC patients with high CTSL-related signature scores displayed increased activity in many important immune-related pathways, including adaptive immune response, immunological response, T-cell receptor signaling pathways, and T-cell activation (**Figure 8B**). Our research suggested that tumor immunity may be closely related to the CTSL signature for HNSCC patients.

Overall, our data show that CTSL is a good indicator of a patient’s prognosis for HNSCC.

## 4 Discussion

A global outbreak of coronavirus infections was brought on by the new human-infecting beta coronavirus known as the severe acute respiratory syndrome coronavirus 2 (SARS-CoV-2) (1). Therefore, to effectively manage SARS-CoV-2, it is crucial to comprehensively examine the factors contributing to COVID-19 susceptibility, the underlying mechanisms of these factors, and implementing measures to prevent infection. It has been established that CTSL is important in coronavirus infection of host cells. Bollavaram et al. revealed that the SARS-CoV-2 spike protein has numerous regions vulnerable to CTSL proteolysis (13). A recent study found that downregulating CTSL expression could reduce the virus’s ability to penetrate host cells (14). An increasing body of evidence suggests that cancer patients are more likely to become severely ill or die from SARS-CoV-2 infection than those without cancer (15–17). In the present study, we assessed the expression levels of viral entry receptors such as CTSL in HNSC cancer tissues since malignant pathology may influence COVID-19 susceptibility and sickness (1, 17).

Our findings provided evidence that HNSC exhibited elevated expression of CTSL. It has been suggested that organs with high CTSL expression may eventually become infected with SARS-CoV-2 (18). Although CTSL expression in HNSC is not as high as in lung cancer, HNSC patients are at significant risk of contracting SARS-CoV-2 infection. The higher susceptibility to infection is probably attributed to the exposure of organs to the external environment, which offers favorable transmission routes for SARS-CoV-2 (19, 20).

In this study, the predictive significance of CTSL for patients with HNSC was assessed using a Cox regression analysis and a prognostic nomogram based on CTSL expression and OS (**Figure 1**). These findings suggest that CTSL may be a potential biomarker for determining the course of HNSC.

A potent tool that can handle the large and complex datasets produced by high-throughput technologies is machine learning used in bioinformatics. It can accurately and quickly analyze biological data, spot trends, and relationships, support drug discovery, enable personalized medicine, and speed up biological research. In the end, machine learning in bioinformatics improves disease understanding, detection, and treatment. This study also applied various machine learning methods, such as random survival forest and Lasso regression, which makes the current study convincing (18, 21).

Moreover, our genomic analysis revealed a correlation between CTSL and mutations in P53, ATRX, and PTEN, and TP53 mutations were associated with poor survival in HNSCC patients and tumor resistance to radiation and chemotherapy clinically (22). Importantly, we found that patients with high expression of CTSL had low IC50 values for 5-fluorouracil, dasatinib, ERK_2440, JAK1_8709, luminespib, and staurosporine, suggesting that CTSL expression was a reliable indicator of therapeutic sensitivity for these potential molecular drugs. Clinical trials have validated the safety and compatibility of these drugs, thereby providing general treatment recommendations for HNSC.

A preliminary overview of CTSL and immune cell infiltration was provided in this study. High CTSL expression in HNSC patient tissues may lead to a deterioration in immune function in patients infected with SARS-CoV-2 because of the relationship between CTSL and the immune response. Furthermore, the infiltration of CD8+ T cells, B cells, CD4+ T cells, neutrophils, macrophages, and dendritic cells in HNSC was associated with the expression of CTSL. Moreover, CTSL was associated with various gene set indicators of different immune cell types. As reported, COVID-19’s poor prognosis is attributed to cytokine storms and inflammatory immune responses (23). The potential role of CTSL in regulating antitumor immunity and its therapeutic significance in HNSC remains unclear. Adaptive immunity is essential for effective viral clearance after SARS-CoV-2 infection. Viral infections and associated chronic inflammatory responses are often associated with cancer manifestation and progression. SARS-CoV-2 may not directly cause cancer, but it may alter the immune landscape and cause adverse outcomes in patients with cancer (24), The immune response of cancer patients may also contribute to the adverse effects of SARS-CoV-2 infection (23). It is possible that CTSL plays a key role in cancer progression and SARS-CoV-2 infection.

There are several limitations and shortcomings to this study. Firstly, the findings heavily rely on bioinformatics analysis, and there is no validation cohort to confirm the results. Additionally, due to the lack of experimental evidence and mechanistic investigations, it is challenging to understand the relationship between SARS-CoV-2 and cancer data and the potential contribution of CTSL. Therefore, more experimental evidence is required.

Moreover, we developed a CTSL-based signature in this study. Patients with low CTSL-based signature scores had higher immune scores, stromal cells, and immune-related pathways, and they responded well to immunotherapy and targeted therapy compared to those with high CTSL-based signature scores. Our results suggest that the CTSL-based signature is a reliable prognostic predictor for HNSCC.

To summarize, we have discussed the clinical and molecular significance of CTSL in HNSC. Our analysis revealed high expression of CTSL in HNSC and its association with poor prognosis and immune cell infiltration. This finding suggests that CTSL may have a biological role in HNSC. Other gene signatures may also have prognostic value for HNSCC, similar to the CTSL-based signature developed in this study. Future studies should investigate these signatures to identify the optimal therapeutic targets for HNSCC.

## Data availability statement

The datasets presented in this study can be found in online repositories. The names of the repository/repositories and accession number(s) can be found within the article/**Supplementary Materials**.

## Author contributions

The study was created and planned by OS and DL. XW and NQ collected the data. FG, MXZ, and MFL carried out the bioinformatic analysis, and FG wrote the manuscript. The statistical analysis was carried out by XW and MQZ under OS’s supervision. The manuscript has been read, carefully amended, and approved by all authors. All authors contributed to the article.
