# Supplementary figures and images for: The analysis of cathepsin L that mediates cellular SARS‐CoV‐2 infection leading to COVID‐19 in head and neck squamous cell carcinoma
Source: Front Immunol. 2023 May 23;14:1156038. doi: 10.3389/fimmu.2023.1156038 (PMC10246451; doi:10.3389/fimmu.2023.1156038)

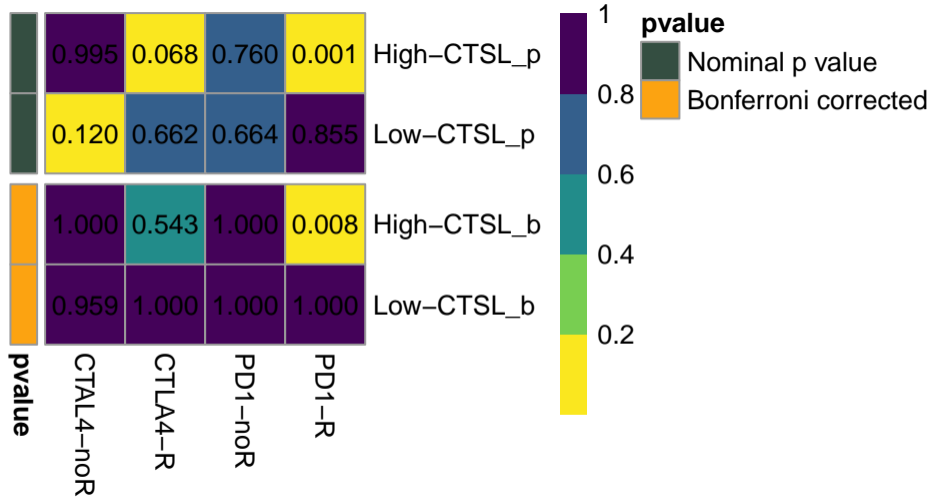

Supplement: Supplementary file 1 [file DataSheet_1.pdf]
